# Does Low-Taper Root Canal Shaping Decrease the Risk of Root Fracture? A Systematic Review

**DOI:** 10.3390/dj10060094

**Published:** 2022-06-01

**Authors:** Francesco Puleio, Giuseppe Lo Giudice, Angela Militi, Ugo Bellezza, Roberto Lo Giudice

**Affiliations:** 1 Department of Biomedical and Dental Sciences and Morphofunctional Imaging, Messina University, 98100 Messina, Italy; francesco.puleio@live.it (F.P.); amiliti@unime.it (A.M.); 2 Department of Dentistry, Sapienza University of Rome, Piazzale Aldo Moro 5, 00185 Rome, Italy; bellezzaugo@gmail.com; 3 Department of Clinical and Experimental Medicine, Messina University, 98100 Messina, Italy; roberto.logiudice@unime.it

**Keywords:** root fracture, endodontic instrument, root canal shaping, microcracks, instrument taper, fracture resistance, root canal preparation

## Abstract

Minimal root-canal preparation has been suggested to reduce the risk of root fracture, but as a result, satisfactory cleaning and shaping do not take place. Large-scale taper instrumentation can contribute to removing infected tissue; however, it may weaken the tooth structure. The aim of this systematic review is to evaluate whether root-canal shaping with low-taper instruments decreases the risk of root fracture, compared to high-conicity shaping. A search was performed on Ovid MEDLINE, PubMed, and the Web of Science. The inclusion criteria were: studies comparing the root fracture resistance of endodontically treated teeth, shaped with low- and high-conicity taper instruments, in human trials, and via in vitro study. The review includes all types of endodontically treated teeth, with various instrument tapers. The scientific search engines produced 328 results. Only 20 of the results were evaluated after screening. Based on the articles analyzed, it is not clear whether a taper difference can determine differences in root fracture resistance. No randomized controlled trial (RCTs) with long follow-ups have been published to date. It must also be taken into account that the in vitro studies do not consider the numerous differences that there are between in vitro and clinical evaluation. The review was registered on the PROSPERO website, with the protocol number CRD42020151451.

## 1. Introduction

Endodontically treated teeth show a higher fracture susceptibility linked to the loss of dentinal structure, especially in the marginal ridges. These aspects are mainly associated with the depletion of the dentinal structure following root-canal treatment [1,2,3]. The literature reports root fracture as being the third most common reason for the extraction of an endodontically treated tooth, mostly affecting the premolars [4].

The literature also reports the ways that root canal treatment may initiate dentinal cracks as a result of thinned dentinal walls, especially in the apical area, which can proceed to complete fractures under functional load [5,6,7,8]; these fractures are more frequent in teeth with curved roots [9,10,11].

Fractures occur when the tensile stress in the root-canal wall exceeds the remaining dentin tensile strength [6,12,13].

Currently, there is no evidence that a specific endodontic treatment or a specific preparation step could be somehow related to root fracture; therefore, investigating the key elements associated with the root canal treatment itself, such as root canal taper, could help clinicians in devising effective treatment protocols that would reduce the likelihood of such an event occurring.

Endodontic treatment is composed of different clinical steps, in particular, canal shaping, aimed at mechanically removing the potential infectious reservoirs to facilitate the cleaning of the endodontic spaces and to create an ideal substrate for root-canal sealing, but which could be linked to microcrack formation and fractures [14].

Minimally invasive endodontics, consisting of smaller-access cavities, minimal root canal preparations with an apical diameter ranging between 0.2 mm and 0.4 mm, and a taper that is strictly below 6% has been suggested to reduce the risk of root fracture [14,15].

This type of root canal preparation is expected to lower the stress concentration sites, leading to a lower incidence of root microcrack formation, but it will not produce satisfactory cleaning and shaping [16,17].

Larger taper instrumentation, in contrast, can contribute to deeper infected-tissue removal, achieving the appropriate irrigant penetration level; however, it may weaken the tooth structure and increase the risk of perforation, ledges, canal transportation, and microcrack formation [18,19,20,21].

This manuscript investigates the clinically relevant topic of the increased fracture susceptibility of endodontically treated teeth, considering that it is not clear whether a larger taper of the endodontic canal could increase the risk of fracture.

The aim of this systematic review is to evaluate if root-canal shaping with low-taper instruments decreases the risk of root fracture compared to high-conicity shaping, using the PICO model: “In human teeth (P), does low-taper root-canal shaping (I), compared to high-conicity shaping (C), decrease the risk of root fracture (O)?”.

## 2. Materials and Methods

### 2.1. Protocol and Registration

The guidelines of the preferred reporting items of systematic reviews and meta-analyses (PRISMA) procedure were followed for the writing of this systematic review [22]. A point-to-point protocol describing the methodology was developed before the writing of this paper. The review was registered in the CRD York website, PROSPERO (protocol number, CRD42020151451).

### 2.2. Search Strategy

The systematic review was carried out on electronic databases, including Ovid MEDLINE, PubMed, and the Web of Science. No search by hand was performed on other databases. The date parameter of the paper collation was set from January 2011 until April 2022.

The following terms and their combinations were searched: (Root fracture) AND (Instrument taper). The keywords were selected to gather and register as much relevant data as possible.

The following focus question was developed, according to the population, intervention, comparison, and outcome (PICO) study design:

“In human teeth (P), does low-taper root-canal shaping (I), compared to high-conicity shaping (C), decrease the risk of root fracture (O)?”.

In agreement with other authors, for this study, outcome tapers below 6% are considered low, while tapers equal to or above 6% are considered high [23,24,25,26]. The review included in vitro and finite element analysis (FEA) studies that compared the root-fracture resistance of dental elements shaped with either low- or high-conicity instruments.

Research comparing the root-fracture resistance of dental elements shaped with identical tools in terms of movement, design, and alloy, but having low and high tapers will be discussed separately.

Only those studies published between January 2011 and April 2022 were considered.

### 2.3. Eligibility Criteria

The full texts of all possibly relevant research papers were chosen, considering the following inclusion criteria:

- Studies comparing the root fracture resistance of endodontically treated dental elements, shaped with low- or high-conicity taper instruments;
- Human trials (randomized controlled trial and clinical trial);
- In vitro studies;
- Finite element analysis.

The exclusion criteria that were considered were:

- Research involving patients with dental diseases linked to reduced chemical/mechanical teeth strength;
- Studies that used only one type of taper;
- Case reports, case series, reviews, and meta-analyses;
- Papers without the full text being available;
- Papers not in the English language.

### 2.4. Risk of Bias Assessment

The evaluation of in vitro studies was set up with a methodological index that uses a checklist for in vitro studies on dental materials (CONSORT). This checklist of items has the purpose of evaluating how the study was designed, analyzed, and interpreted, and uses 14 domains [27].

Item 1. Abstract:

Structured summary of the trial’s design, methods, results, and conclusions.

Item 2. Background and objectives:
  Item 2a. Scientific background and explanation of the rationale.
  Item 2b. Specific objectives and/or hypotheses.

Item 3. Intervention:

The intervention for each group, including how and when it was administered, with sufficient detail given to enable replication.

Item 4. Outcomes:

Completely defined, pre-specified primary and secondary measures of the outcome, including how and when they were assessed.

Item 5. Sample size:

How the sample size was determined.

Item 6. Randomization and sequence generation:

The method used to generate the random allocation sequence.

Item 7. Allocation concealment mechanism:

The mechanism used to implement the random allocation sequence (for example, sequentially numbered containers), describing any steps taken to conceal the sequence until intervention was assigned.

Item 8. Implementation:

Who generated the random allocation sequence, who enrolled the patients, and who assigned the patients to intervention?

Item 9. Blinding:

If conducted, who was blinded after assignment to the intervention process (for example, care providers or those assessing the outcomes), and how this was achieved.

Item 10. Statistical methods:

The statistical methods used to compare the groups for primary and secondary outcomes.

Item 11. Results, outcomes, and estimation:

For each primary and secondary outcome, the results for each group and the estimated size of the effect and its precision (for example 95% confidence interval).

Item 12. Discussion and limitations:

Trial limitations, addressing the sources of potential bias, imprecision, and, if relevant, the multiplicity of analyses of other information.

Item 13. Funding:

Sources of funding and other support (for example, the suppliers of drugs) and the role of the funders.

Item 14. Protocol:

Where the full trial protocol can be accessed, if available.

The risk of bias was conducted at the study level.

## 3. Results

### 3.1. Study Selection

Two researchers from Messina University (F.P., R. Lo G.) conducted the same literature search independently, and, in cases of discrepancies in the results, consulted a third senior researcher (G. Lo G.) for each phase (initial screening, the eligibility for final inclusion, data extraction and analysis, and quality assessment).

**Figure 1:**
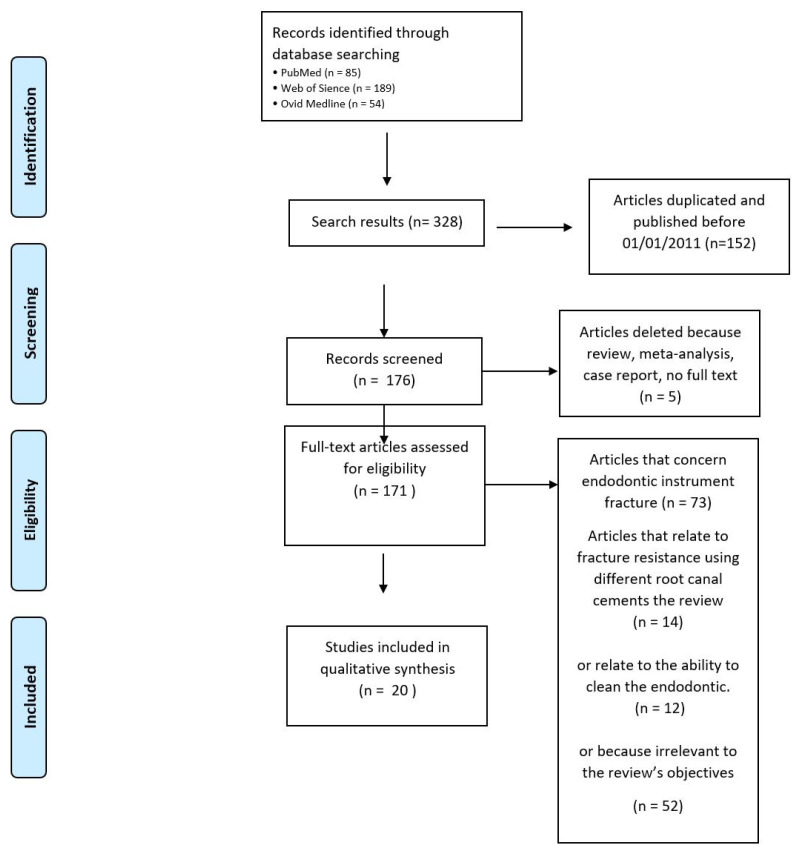
PRISMA flow chart.

The scientific search engines produced 328 results. Research duplicates and studies published before 1 January 2011 were not included, obtaining a total number of 176 studies. In total, 5 articles were not included because the full text was not available or the article was a review, meta-analysis, or case report. After the first selection, 171 studies underwent a full-text examination. Of these 171 articles, 73 were discarded because they evaluated endodontic instrument fracture, while 14 were not included as they studied fracture resistance using different root-canal cements; 12 were discarded since they analyzed the ability to clean an endodontic space shaped with a different taper, while 52 were discarded because they were not related to the review’s objectives. In all, 20 studies were included in this review [28,29,30,31,32,33,34,35,36,37,38,39,40,41,42,43,44,45,46,47] (Figure 1). The included papers are listed in Table 1. Among the 20 studies included in the revision, 6 of these compare the results obtained using different tapers of the exact same instrument.

### 3.2. Risk of Bias

Table 2 presents the risk of bias in the in vitro studies.

## 4. Discussion

All the research examined in this review is of in vitro studies and FEA analysis. Table 2 shows the bias risk for the articles included, which was evaluated as high due to the absence of a blinded investigator and of random sequence generation, potentially introducing a selection bias.

To evaluate the effects of the different shapes of taper, the authors used various devices and methods, such as fracture load, micro-CT, radicular section observation at different magnifications, and finite element analysis (FEA). The FEA method has been successfully used in endodontics, where it has provided some valuable insights into the fracture mechanisms [48].

The studies selected evaluated the root resistance, analyzing:

- The presence of/variations in the number of microcracks observable by micro-CT or under a microscope.
- Variations in the fracture resistance to the fracture load test.
- Variations in the stress distribution, using finite element analysis.

The study design of the papers that were found and are included in the review is highly heterogeneous. The majority of the authors analyzed the resistance variation related to radicular fracture, while others analyzed radicular microcrack formation. Therefore, in addition to the primary outcome, “decrease in fracture resistance”, which was evaluated following the PICO scheme, “microcrack formation” has been evaluated as a secondary outcome.

The in vitro studies that were included do not agree among themselves when comparing their results and evaluating the primary outcome. Some authors show how increasing the root canal taper does not produce root resistance reduction [32,34,36,45]. Others, instead, show how increasing the root canal taper decreases the root resistance [30,31,37,38].

Munari et al. instead used finite element analysis to show that as the root canal diameter increases, there will be a bigger circumferential area for distributing the contact pressure, leading to lower fracture-causing stresses [33]. This result is contrary to the results of the other authors.

The study by Cicek et al. shows how the group contoured using the Protaper Next x4 (40/0.06) had a wall resistance that was significantly greater than the other groups included in the same study, in which samples had been shaped with a file with a less (Twisted File 40/0.04), equal (Protaper Universal F4 40/0.06), or greater (WaveOne Large 40/0.08) taper; the group shaped using Mtwo (40/0.06) showed a significantly lower fracture resistance [43]. The instruments used in the papers that are included in this systematic review are all extremely different in characteristics such as instrument alloy, movement type, and blade design; therefore, it is not possible to isolate the “taper” parameter from the results.

Whereas root fracture was presented by some authors as an outcome, some of the included studies dealt with the development of microcracks in extracted teeth. According to some authors, these microcracks could ultimately lead to root fractures [49,50,51,52,53]. According to a recent narrative review, however, endodontic shaping may not be the cause of microcrack formation. In fact, these defects can be observed in extracted and stored teeth and may be due to procedural errors in the preparation of the experimental samples, rather than merely being due to the shaping [54]. Moreover, the studies that analyze microcrack formation showed discordant results, even when compared to each other [28,34,39,40,41,42,44,46,47].

Arias et al. showed how increasing the root canal taper does not determine an increased frequency of microcrack formation. [39,46]. Other authors instead showed how increased root canal taper is linked to an increase in microcrack formation [40,41,42].

Jamleh et al., instead, showed that the root resistance variation is not related to the differences in instrument taper, but is instead attributable to a different instrument movement (continuous rotation and reciprocating movement); this study compared the formation of microcracks in shaped elements with continuous rotation or with reciprocating movement [44]. Several studies have shown that reciprocating movement was also responsible for the strain decrease on dentinal walls while the instrument moved into the root canal [55,56]. Their findings suggest that, in terms of the occurrence of microcracks, the instrumentation motion is more important than the file taper, which does not have a specific effect.

Two of the studies included in this review show ambiguous results [34,47].

1. The paper by Hin et al. compares the incidence of root dentin cracks after root-canal preparation with hand files (40/0.05), a self-adjusting file (SAF), the ProTaper Universal F4 (40/0.06), and Mtwo (40/0.04) [47]; in this study, the group in which manual shaping was used (40/0.05) showed a significantly lower number of microcracks than the group shaped using the ProTaper Universal F4 (40/0.06) and the one shaped with the Mtwo (40/0.04). The reason why the group where shaping was performed with a 0.05 taper shows a lower number of microcracks could be related to the type of instrumentation used. Other authors have shown that manual shaping causes less stress on the root canal walls, compared to rotative instruments [48].
2. The study by Aksoy et al. states that the Protaper Universal F2 (25/0.08) system significantly increased the percentage rate of microcracks compared with the XP (25/0) and Reciproc Blu (25/0.08) groups [34]. In this study, the increase in microcracks using a Protaper Universal 25/0.08, compared to another system with the same apical diameter and same instrument taper, is related to the greater stress transmitted to dentin caused by the continuous rotative movement compared to reciprocating movement [55,56,57].

The root canal instrumentation technique known as SAF has been analyzed in three research papers included in this review [39,45,47]. The SAF technology uses a hollow, compressible NiTi file with no central metal core, through which continuous irrigant flow is provided throughout the procedure, avoiding the unnecessary and excessive removal of sound dentin [58,59].

Kfin et al. and Hin et al. state, in their respective studies, that when microscopically observing the shaped specimens, the SAF caused fewer microcracks than the Protaper Universal F3 (30/0.09), WaveOne Primary (25/0.08), Protaper Universal F4 (40/0.06) and Mtwo (30/0.04) [40,47]. The absence of a central metal core in the SAF file and its extreme compressibility might explain the difference between this file and the other rotary or reciprocating file systems [45,60,61,62].

The differences in the results of the studies included in this revision are, therefore, attributable to many factors and are not related to the taper of the instrumentation used, but instead are probably related to:

- The difference in the preparation motion [48,55,56];
- The cross-sectional profile and blade design [10,63,64];
- The type of alloy of the instrument used [54,64,65,66].

Among the 20 studies included in this revision, 6 compare the results obtained using the same tools from the same manufacturer in the shaping phase but with different tables. In this way, it is possible to exclude those variables already discussed (preparation motion, blade design, and the type of alloy used in the instrument). However, their results are not in agreement. Some authors report that a low taper increases the resistance to fracture, compared to teeth shaped with an instrument with a greater taper [30,31,33,37]. Other authors report that there is no correlation [32,36]. However, the small number of these studies does not allow us to reach clear conclusions.

### Limitations

The first limitation of the in vitro studies considered is the high risk of bias, due to the absence of a blinded investigator and the random sequence generation methodology.

The second limitation is linked to the heterogeneous sample of the studies analyzed in this review. Some authors evaluated incisors, while others evaluated premolars; others evaluated molars, while others merely evaluated the roots. Therefore, there is a lack of standardization.

No RCTs with long follow-ups have been published to date. A prospective clinical study design for the investigation of this parameter is impractical, as the evaluated outcome may take years to occur and could be linked to any kind of oral and systemic modification in the patient; moreover, there is a lack of a standardized and accurate diagnostic tool with which to diagnose problems. The retrospective extraction of data from clinical studies presents several limitations, as well; isolating this parameter from the patient’s other tooth- and treatment-related risk factors is very difficult, and confounding is highly likely. Therefore, systematically reviewing the in vitro studies seems a justifiable way to shed more light on this topic; despite the well-known limitations concerning the extrapolation of findings to clinical practice, the internal validity of in vitro studies can be adequately addressed. It must also be considered that in vitro studies do not take into account the numerous differences that there are between in vitro and clinical evaluation. Any further papers should propose standardization in the clinical protocol to evaluate root fractures and to distinguish them from the microcracks, evaluating if, and how, one type could evolve into the other; moreover, more papers are needed to evaluate the mechanical behavior of the different teeth, as related to their different root shapes and force distribution. Due to the nature of the research, an FEA study could be used to speed up the research process and provide a more standardized and scientifically reliable analysis.

## 5. Conclusions

Based on the articles analyzed in this systematic review, it is not clear whether a difference in taper angle can determine differences in root fracture resistance. The studies included in this review do not agree in terms of their results. In addition, all the included studies are in vitro studies. Therefore, it is not possible to provide a clinical recommendation regarding the use of endodontic instruments with a high or a low taper.

Further studies are necessary to develop a unique protocol, using instruments with exactly the same characteristics (movement, design, alloy, etc.) but changing only the taper angle of the instrument.

## Figures and Tables

**Table 1:** PRISMA flow chart. The characteristics of the included studies.

Author	Object of Research	Taper	Study Design and Evaluation Methods	Result
Eliasz W. 2022 [28]	80 single-rooted teeth	Co, 25/0.06 PTN 25/0.08 WO 35/0.04 TF	Randomized controlled trial Observation with microscope at 25× magnification	No significant differences were observed among experimental groups.
Lin G. S. S. 2022 [29]	80 mandibular premolars	Co, 25/0.04 TP, 25/0.04 HyF, 25/0.06 Tg, 25/0.06 Zf.	Randomized controlled trial Fracture load	The fracture strengths of the 25/0.04 group were found to be significantly higher than in the 25/0.06 group.
Kılıç Y. 2021 [30]	55 mandibular molars	Co, 25/0.04 VDWr, 25/0.06 VDWr, 30/0.04 VDWr, 30/0.06 VDWr.	Randomized controlled trial Fracture load	The fracture strengths of the 25/0.04 group were found to be significantly higher than in the other groups.
Doganay Y. 2020 [31]	84 mandibular incisors	Co, 25/0.04 K3, 25/0.06 K3, 25/0.08 K3, 30/0.04 K3, 30/0.06 K3, 30/0.08 K3.	Non-randomized controlled trial Fracture load	Significant differences were found between 25/0.04 and 25/0.08; 30/0.04 and 30/0.08; and 25/0.08 and 30/0.04.
Tian S. Y. 2019 [32]	100 human permanent mandibular premolars with a straight single canal	Co, Hand-file: 40/0.05, 45/0.05, 50/0.05, 55/0.05, 60/0.05, 40/0.10, 40/0.15, 45/0.10, 45/0.15.	Randomized controlled trial Fracture load	No significant differences in the fracture modes were detected among the 10 groups.
Munari L. S. 2019 [33]	36 single-rooted lower premolars	35/0.02 K3, 35/0.04 K3, 35/0.06 K3	Analytics cohort study Finite element analysis	Both analytical and FE solutions showed a positive linear relationship between the fracture load and the enlarged root-canal diameter.
Aksoy C. 2019 [34]	30 mandibular first and second molars	25/0 XP, 25/0.08 RB, 25/0.08 PTU	Non-randomized controlled trial Micro-CT	No new dentinal microcracks were observed in the XP and RB groups. The PTU system significantly increased the percentage rate of microcracks, compared with the preoperative specimens.
Krikeli E. 2018 [35]	58 maxillary canines	Co, 40/0.02 Hf, 40/0.04 MT, 40/0.06 MT	Randomized controlled trial Fracture load	Only 40/0.06 MT Vs Co was statistically significant.
Zogheib C. 2018 [36]	60 maxillary premolars	30/0.04 IR, 30/0.06 IR	Non-randomized controlled trial Fracture load	No statistically significant difference was registered.
Sabeti M. 2018 [37]	30 distobuccal roots of maxillary molars	25/0.04 TF, 25.0.06 TF, 25/0.08 TF	Randomized controlled trial Fracture load	The 0.04 taper and 0.06 taper groups did not significantly differ, but both groups differed significantly from the 0.08 taper group.
Askerbeyli S. 2017 [38]	1 two-rooted premolar and 3 single-rooted premolars	Co, 30/0.04 HS, 30/0.06 RS, 30/0.09 PTU	Analytics cohort study Finite element analysis	The intact models exhibited the lowest stress values, followed by the 30/0.04 model, while 30/0.09 exhibited the highest stress values.
Kfir A. 2016 [39]	80 extracted maxillary first premolars two-rooted	Co, 30/0.09 PTU, 25/0.08 WO, SAF	Randomized controlled trial Observation with a microscope at 20× magnification	The differences between both the PTU treated and the WO groups, compared to the SAF treated group, were significant. No difference was seen between 30/0.09 and 25/0.08.
Ceyhanli. K. T. 2016 [40]	30 mandibular molars	30/0.09 PTU, 30/0.04 IR, 30/0.04 SS	Non-randomized controlled trial Micro-CT	The PTU system generated more post-instrumentation dentinal microcracks.
Li S. 2015 [41]	60 molars	Co, 25/0.08 PTU, 25/0.08 WO, 25/0.06 PTN	Randomized controlled trial Observation with a stereomicroscope at 60× magnification	The 25/0.06 PTN induced fewer dentinal microcracks during the root canal procedures in severely curved root canals, compared with the PTU and WO systems.
Karatas E. 2015 [42]	75 central incisors	Control, 25/0.08 PTU, 25/0.06 PTN, 25/0.08 WO, 25/0.06 TFA	Non-randomized controlled trial Observation with a stereomicroscope at 25× magnification	The PTN and TFA systems produced significantly fewer cracks than the PTU and WO systems.
Cicek, E. 2015 [43]	72 mandibular first premolar	40/0.06 PTU, 40/0.06 PTN, 40/0.08 WO, 40/0.04 TF, 40/0.06 MT, 40/0.06 RS	Non-randomized controlled trial Fracture load	The PTN group was the most resistant to fracture, while the MT group was the least resistant. Resistances between the WO Group and RS Group were similar.
Jamleh A. 2014 [44]	25 mandibular premolars	Co, 40/0.06 PTU, 40/0.08 WO	Randomized controlled trial Micro-CT	Fewer microcracks were found after instrumentation with a 40/0.08 WO.
Capar I. D. 2014 [45]	50 mandibular premolars	Co, 40/0.06 PTU, SAF	Randomized controlled trial Fracture load	The differences were not statistically significant.
Arias A. 2014 [46]	18 lower incisors	Co, 25/0.04 manual PF, 25/0.08 WO	Randomized controlled trial Photo observation at 25× and 40× magnification	There were no significant differences in the incidence of microcracks between all groups.
Hin E. A. 2013 [47]	100 mandibular premolars	Co, 40/0.05 Hf, 40/0.06 PTU, 40/0.04 MT, SAF	Non-randomized controlled trial Photo observation at 12× magnification	The Hf group did not show any dentinal cracks. The PTU and MT caused more cracks than Hf, but SAF did not.

Co: control; VDWr: VDV Rotate; TP: T-Pro; HyF: HyFlex CM; Tg: TG6; Zf: ZenFlex; PTG: ProTaper Gold; Hf: hand file; XP: XP-endo Shaper; RB: Reciproc Blu; PTU: ProTaper Universal; MT: Mtwo; IR: iRaCe; TF: twisted files; HS: HeroShaper; RS: Revo; WO: WaveOne; SAF: self-adjusting file; SS: Safesider; PTN: ProTaper Next; TFa: TF Adaptive; PF: ProFile GT.

**Table 2:** Summary of the bias risk for in vitro studies, according to CONSORT.

Item	Eliasz W. 2022 [28]	Lin G. S. S. 2022 [29]	Kılıç Y. 2021 [30]	Doganay Y. 2020 [31]	Tian S. Y. [32]	Munari L. S. [33]	Aksoy C. [34]	Krikeli E. [35]	Zogheib C. [36]	Sabeti M. [37]	Askerbeyli S. [38]	Kfir A. [39]	Ceyhanli. K. T. [40]	Li S. 2015 [41]	Karatas E. [42]	Cicek, E. [43]	Jamleh A. [44]	Capar I. D. [45]	Arias A. [46]	Hin E. A. [47]
1 Abstract	Yes	Yes	Yes	Yes	Yes	Yes	Yes	Yes	Yes	Yes	Yes	Yes	Yes	Yes	Yes	Yes	Yes	Yes	Yes	Yes
2a Background and objectives	Yes	Yes	Yes	Yes	Yes	Yes	Yes	Yes	Yes	Yes	Yes	Yes	Yes	Yes	Yes	Yes	Yes	Yes	Yes	Yes
2b Background and objectives	Yes	Yes	Yes	Yes	Yes	Yes	Yes	Yes	Yes	Yes	Yes	Yes	Yes	Yes	Yes	Yes	Yes	Yes	Yes	Yes
3 Intervention	Yes	Yes	Yes	Yes	Yes	Yes	Yes	Yes	Yes	Yes	Yes	Yes	Yes	Yes	Yes	Yes	Yes	Yes	Yes	Yes
4 Outcomes	Yes	Yes	Yes	Yes	Yes	Yes	Yes	Yes	Yes	Yes	Yes	Yes	Yes	Yes	Yes	Yes	Yes	Yes	Yes	Yes
5 Sample size	No	No	No	Yes	No	No	No	No	No	No	No	No	No	No	No	No	No	No	No	No
6 Randomization: Sequence generation	Yes	Yes	Yes	No	Yes	No	No	Yes	No	Yes	no	Yes	No	Yes	No	No	Yes	Yes	Yes	No
7 Allocation concealment mechanism	No	No	No	No	No	No	No	No	No	No	No	No	No	No	No	No	No	No	No	No
8 Implementation	No	No	No	Yes	No	No	No	No	No	No	No	No	No	No	No	No	No	Yes	No	No
9 Blinding	No	No	No	No	No	No	No	No	No	No	No	Yes	No	No	No	No	No	No	Yes	No
10 Statistical methods	Yes	Yes	Yes	Yes	Yes	No	No	Yes	No	No	No	No	No	Yes	Yes	Yes	Yes	Yes	Yes	No
11 Results, outcomes, and estimation	Yes	Yes	Yes	Yes	Yes	Yes	Yes	Yes	Yes	Yes	Yes	Yes	Yes	Yes	Yes	Yes	No	No	Yes	Yes
12 Discussion Limitations	Yes	Yes	Yes	Yes	No	No	Yes	No	No	No	No	No	Yes	yes	No	No	Yes	Yes	Yes	No
13 Other information Funding	Yes	No	No	No	No	No	No	No	Yes	No	No	No	No	No	No	No	No	No	No	No
14 Protocol	Yes	Yes	Yes	Yes	Yes	Yes	Yes	Yes	Yes	Yes	Yes	Yes	Yes	Yes	Yes	Yes	Yes	Yes	Yes	Yes

## Data Availability

The data can be obtained upon request to the corresponding author.
